# Assessment of Influenza Testing Distribution in the United States for the 2021-2022, 2022-2023, and 2023-2024 Influenza Seasons: Online Cross-Sectional Study

**DOI:** 10.2196/76459

**Published:** 2025-08-26

**Authors:** Autumn Gertz, Juliana B Sopko, Christopher Remmel, Benjamin Rader, John S Brownstein

**Affiliations:** 1 Boston Children's Hospital, 300 Longwood Ave, Boston, MA, 02215, United States, 1 6173556000; 2 Harvard Medical School, Boston, MA, United States

**Keywords:** influenza, flu testing, syndromic surveillance, health survey, influenza-like illness

## Abstract

**Background:**

Effective surveillance of seasonal influenza is crucial to understanding disease burden and impact. Traditional surveillance accounts for those who interact with the health care system, including those who are testing for diseases like influenza. However, care seeking and testing are not as common with influenza and can lead to bias. Better understanding who is being captured by current surveillance methods can help further knowledge around influenza and identify areas of improvement in surveillance, disease mitigation, and intervention efforts.

**Objective:**

This study aimed to examine who is testing for influenza amongst a United States representative survey population, across three seasons influenza seasons spanning 2021 to 2024.

**Methods:**

Outbreaks near me (ONM) is a participatory surveillance system that, in partnership with SurveyMonkey, conducted a web-based, weekly cross-sectional survey. ONM Survey data from three influenza seasons was used in this study: 2021‐2022, 2022‐2023, and 2023‐2024. Tested for influenza was defined as a “yes” response to “In the past 30 days, have you been tested for influenza (flu)?” Descriptive proportions applying survey weights reflecting US census targets were produced to understand which demographic groups were testing for influenza. A weighted multivariate logistic regression was conducted for influenza testing by income, adjusting for other demographics and COVID-19 testing. Descriptive proportions and multivariate regressions were conducted by influenza season.

**Results:**

In total, 940,172 responses were collected, with similar amounts in 2021‐2022 (n=335,964) and 2022‐2023 (n=334,584), and slightly less in 2023‐2024 (n=269,624). Generally, low levels of influenza testing were reported in each season at 4.2%, 9.1%, and 8.9%, respectively. Weighted proportions of those who tested for influenza only and no other diseases (like COVID-19) were even lower (0.4%, 971/335,964; 1.5%, 4,382/334,584; and 2.0%, 4579/269,624; respectively). Broadly, those who had lower income tested for influenza at progressively higher proportions. A similar trend was observed season to season with education level as well. Across the 3 observed influenza seasons, lower household annual income (under US $15,000) was associated with higher odds of testing for influenza (2021‐2022: adjusted odds ratio [AOR] 1.41, 95% CI 1.34‐1.48; 2022‐2023: AOR 1.42, 95% CI 1.35‐1.49; 2023‐2024: AOR 1.25, 95% CI 1.18‐1.34), while those with higher incomes (over US $150,000) were less likely to have been tested for influenza (2021‐2022: AOR 0.64, 95% CI 0.55‐0.86; 2022‐2023: AOR 0.82, 95% CI 0.73‐0.91; 2023‐2024: AOR 0.66, 95% CI 0.56‐0.76).

**Conclusions:**

Within this study population, individuals who fall within lower-income brackets tested for influenza more than their higher-income counterparts. In all 3 seasons spanning 2021‐2024, lower income was associated with higher proportions of influenza testing and an increased likelihood of having tested for influenza in the past 30 days. These trends suggest that populations that may experience more barriers to care are not only accessing influenza testing but doing so differently than groups that historically access care.

## Introduction

Effective surveillance of respiratory illnesses is crucial to early outbreak detection and robust response efforts [1]. In the United States, surveillance of respiratory disease, such as influenza, has largely been conducted via encounters with the health care system [2]. Laboratory-confirmed influenza cases, outpatient influenza-like illness (ILI), and mortality data help public health researchers gauge the burden of influenza [2]. However, variable access to medical care and health care usage suggests that traditional surveillance is only capturing a part of the population [3]. Influenza severity and burden have been observed to vary across demographics, particularly that racial and ethnic minorities continue to experience higher hospitalization rates for influenza compared to White people [4]. Influenza vaccination rates have also varied across racial and ethnic groups in the United States [4].

During the COVID-19 pandemic, a robust and rapid expansion of testing occurred. Throughout the pandemic, governments turned to large-scale testing efforts to help mitigate the spread of COVID-19 [5]. Early surveillance efforts relied heavily upon traditional data streams like testing centers and hospital data [6]. However, as the pandemic progressed, changes such as the introduction of at-home rapid antigen tests impacted testing, care-seeking behaviors, and surveillance strategy [7,8]. An uptick in at-home testing for COVID-19 was observed, particularly among individuals who identified as White, had higher education attainment, and higher annual income, despite efforts to increase availability and access [9,10]. While such efforts did not explicitly include other respiratory illnesses like influenza, they may have led to secondary changes. For example, test administration within hospitals evolved over the pandemic to assess incidence of pathogens causing acute respiratory illnesses [11]. More recently, multiplex assays have been approved and used to assess single samples for both influenza and COVID-19 [12,13].

In addition to advancements like at-home and multiplex testing, digital disease surveillance methods like participatory surveillance can augment traditional disease surveillance [7,8,14,15]. Outbreaks near me (ONM) is an online participatory syndromic surveillance system that allows for the general public to report disease and health-related information in the United States [16]. ONM enables near-real-time collection and aggregation of data related to disease burden, vaccination coverage, and symptomatology [16]. In addition, participants are asked to answer questions related to age, gender, race, income, and occupation.

In this study, we leverage the previously validated ONM cross-sectional survey to assess the population testing for influenza in the 2021‐2022, 2022‐2023, and 2023‐2024 influenza seasons [10,17]. Specifically, we assess which demographic groups were likely to get tested for influenza and examine if there were any changes in the seasons following the COVID-19 pandemic. ONM is well situated to capture and evaluate any trends and changes to influenza testing, to further the understanding of the impacts and burden of influenza. Here we use the ONM survey to assess who is testing for influenza and identify which populations are represented, or not, in existing influenza surveillance.

## Methods

### Survey Population

The ONM survey was deployed on the SurveyMonkey digital platform. Participants were recruited via end page river sampling, a sampling method that prompts an optional survey after completion of an unrelated survey on SurveyMonkey [18]. With over two million daily visitors to SurveyMonkey, the survey is able to capture a random sample of US residents, collecting between 5000‐15,000 responses per week. Recruitment excludes those younger than 13 years due to sampling and privacy concerns. An IP address is used to mitigate the same individual from being prompted to complete the survey more than once [18]. The survey collected robust demographic data via multiple choice and multiselect questions. The data collected via this survey have been previously validated in other studies [10,17]. Data from the 2021‐2022, 2022‐2023, and 2023‐2024 influenza seasons are included in this analysis. A testable version of the survey from the 2023‐2024 season is available [19]. Influenza seasons are defined as Morbidity and Mortality Weekly Report (MMWR) week 40 (approximately October 1) through March 31, with exact dates of collection in Table S1 in Multimedia Appendix 1. Influenza seasons were truncated to March 31, rather than MMWR week 20, for consistency as data collection ended March 31, 2024.

### Analysis

Respondents were asked “In the past 30 days, have you been tested for influenza (flu)?” with participants being able to select either “yes” or “no.” A series of demographic questions were asked, and we collapsed the self-reported industry responses into 2 categories, health care workers and non–health care workers. “In the past 30 days, have you been tested for COVID-19?” was captured as well. Self-reports of at-home tests are included in these “yes” responses. For descriptive analyses, we assessed 2 influenza testing groups: tested for influenza and only tested for influenza. Tested for influenza included anyone who answered “yes” to “In the past 30 days, have you been tested for influenza (flu)?” Those only tested for influenza comprised a subset of those tested for influenza, which excluded anyone who also reported having been tested for influenza and excluded anyone who also reported “yes” to testing for COVID-19 as well. This group does not exclude anyone who was tested for respiratory illnesses beyond COVID-19. An inverse probability of survey selection weighting scheme targeting the US census estimated population was applied to all results in the study [10,17,18,20]. Influenza positive results were defined as a response of “positive” to “What were the results of your influenza (flu) test?” A weighted multivariate logistic regression model was conducted for each influenza season to assess the likelihood of having been tested for influenza. The models assessed the likelihood of having been tested for influenza in the past thirty days by income, adjusting for age, gender, race, education level, health insurance type, health care worker status, and having been tested for COVID-19 in the past 30 days. Descriptive proportions and regression models were assessed for each influenza season, and not across the study period in its entirety. RStudio (version 2024.12.1+563; Posit) was used to conduct the analyses.

### Ethical Considerations

This study was approved by Boston Children’s Hospital institutional review board for secondary use of data and received a waiver of informed consent (IRB-P00023700). Participation in the survey was voluntary and participants were not compensated. Limited identifiable data were collected in the study, and all data used were deidentified.

## Results

### Survey Population

During the study period, a total of 940,172 responses were collected. The weighted demographic breakdowns of the 2021‐2022, 2022‐2023, and 2023‐2024 influenza seasons were similar and were similar to the US population and were thus deemed representative [20]. In all 3 seasons, more respondents identified as female than as male (Table 1). Across all seasons, the largest proportion of respondents was the group aged 65 years and older (19.3%, 69,893/335,964; 19.5%, 60,415/334,584; and 19.5%, 40,477/269,624; respectively); the smallest proportion was of those younger than 18 years (7.1%, 4457/335,964; 6.5%, 3745/334,584; and 7.1%, 2997/269,624; respectively). Across all seasons, the largest proportion of respondents was of those who completed high school or earned their General Educational Development diplomas (33.3%, 57,899/335,964; 33.2%, 51,033/334,584; and 32.8%, 36,531/269,624; respectively), and the smallest proportion was of those who did not complete high school (8.9%, 10,668/335,964; 8.7%, 9871/334,584; and 9.4%, 7893/269,624; respectively). The majority of respondents received insurance via a plan through their spouse, parent, or employer (39.8%, 151,845/335,964; 36.9%, 99,236/334,584; and 35.0%, 54,920/269,624; respectively), and the smallest proportion of participants received insurance through TRICARE (1.9%, 6333/335,964; 1.9%, 4737/334,584; and 1.8%, 2672/269,624; respectively). The complete results of weighted proportions for industry are available in Table S2 in Multimedia Appendix 1.

**Table 1.** Weighted demographic characteristics of the study population across 3 influenza seasons. An online cross-sectional study of US residents 13 and older was carried out to assess self-reported demographics and influenza testing in the last 30 days for the 2021‐2022 (n=335,964), 2022‐2023 (n=334,584), and 2023‐2024 (n=269,624) seasons. This table displays the demographics of the study population. Proportion uses an inverse probability of survey selection weighting scheme targeting the US census. Numbers are unweighted and reflect the number of individuals in each demographic group during each influenza season.

Demographics		Season^a^					
		2021‐2022 (N=335,964)		2022‐2023 (N=334,584)		2023‐2024 (N=269,624)	
		N	Weighted %	N	Weighted %	N	Weighted %
Gender							
Male		129,233	47.1	100,980	47.1	66,401	47.1
Female		200,684	50.6	160,850	50.8	105,135	50.6
Not listed or nonconforming		6047	2.3	5276	2.1	4094	2.2
Race							
White		217,482	62.1	160,169	61.8	102,610	61.7
Black		46,759	16.7	39,214	16.9	26,417	17.1
Hispanic		43,561	12.5	35,636	12.6	23,210	12.5
Asian		16,029	5.7	12,326	5.4	9119	5.4
No Answer		12,133	3.0	87,239	3.3	108,268	3.3
Education							
Did not complete high school		10,668	8.9	9871	8.7	7893	9.4
High school or General Educational Development diploma		57,899	33.3	51,033	33.2	36,531	32.8
Associate’s degree		32,592	9.1	25,602	9.5	16,269	9.4
Some college		66,617	19.5	51,577	19.4	33,148	19.2
College graduate		91,664	18.2	70,289	18.3	44,833	18.3
Postgraduate degree		76,524	10.9	58,734	10.9	36,956	10.9
Age (years)							
13‐17		4457	7.1	3745	6.5	2997	7.1
18‐24		21,420	11.2	16,655	11.1	11,628	10.9
25‐34		43,489	16.7	33,772	16.8	22,153	16.7
35‐44		56,378	15.5	44,276	15.6	28,582	15.5
45‐54		67,123	14.9	51,021	15.0	33,070	14.9
55‐64		73,204	15.4	57,222	15.5	36,723	15.5
65 and older		69,893	19.3	60,415	19.5	40,477	19.5
Income (US $)							
Under $15,000		30,200	12.8	25,095	13.4	16,376	14.2
Between $15,000 and $29,999		35,390	13.2	26,565	12.9	16,002	12.4
Between $30,000 and $49,999		46,445	15.4	34,833	15.4	20,741	15.3
Between $50,000 and $74,999		50,944	15.1	36,881	15.0	22,096	15.1
Between $75,000 and $99,999		42,361	11.5	29,940	11.3	17,337	11.0
Between $100,000 and $150,000		52,730	12.9	37,042	12.9	21,364	12.9
Over $150,000		55,466	12.2	28,954	12.2	22,557	12.2
No Answer		22,423	6.9	105,274	6.9	133,151	7.0
Insurance							
Plan through your spouse, parent, or employer		151,845	39.8	99,236	36.9	54,920	35.0
Other (please specify)		20,200	6.7	8921	3.8	—^b^	—
Plan you purchased yourself		37,674	10.3	27,863	10.5	16,670	10.5
Medicare		67,835	21.5	56,337	23.1	35,013	23.3
Medicaid or Medical		26,810	9.9	24,050	11.7	15,039	11.9
TRICARE		6333	1.9	4737	1.9	2672	1.8
Not covered by health insurance		19,231	7.7	13,817	7.1	9405	8.0
No Answer		6034	2.2	99,623	5.1	135,905	9.5
Industry							
Non–health care^c^		76,586	78.7	133,084	79.0	177,869	79.9
Health care		25,770	21.3	44,387	21.0	57,672	20.1

a Please refer to Table S1 in Multimedia Appendix 1 for the specific date ranges of the 2021-2022, 2022-2023, and 2023-2024 seasons.

b Not applicable.

c Non–health care is a condensed group that consists of all occupations except health care. To see the complete breakdown of non–health care occupations, refer to Table S2 in Supplement Table 2Multimedia Appendix 1.

### Influenza Testing

During the 2021‐2022 season, 4.2% (11,733/335,964) of the population had tested for influenza and 0.4% (971/335,964) had only tested for influenza. Slightly more tested during 2022‐2023 at 9.1% (26,875/334,584) and 1.5% (4382/334,584) only tested for influenza. In the 2023‐2024 season, 8.9% (21,809/269,624) of the population tested for influenza and 2.0% (4579/269,624) had only tested for influenza. The proportion of individuals who had not completed high school and tested for influenza and only tested for influenza was higher than the other educational groups. Those who made under $15,000 reported more testing in both tested for influenza (7.1%, 2002/335,964; 15.0%, 3640/209,646; and 14.2%, 2319/269,624; respectively) and only tested for influenza (0.8%, 217/335,964; 2.3%, 536/334,584; and 2.7%, 426/269,624; respectively) than higher-income groups (Table 2). Overall, the proportion of individuals with Medicaid or Medical tested for influenza (6.7%, 1659/335,964; 14.4%, 3357/334,584; and 13.2%, 1985/269,624; respectively) and only tested for influenza (0.6%, 130/335,964; 2.1%, 487/334,584; and 2.6%, 376/269,624; respectively) at higher levels (Table 2). There was an observed increase in the proportion of those who did not complete high school and who only tested for influenza (truncated due to low numbers: 2.3%, 251/334,584 and 3.3%, 252/269,624) from 2021 to 2024 (Table 2). Table S3 in Multimedia Appendix 1 contains influenza testing among all the industry options.

**Table 2.** Weighted demographic characteristics of individuals who tested for influenza and only tested for influenza across 3 seasons. An online cross-sectional study of US residents aged 13 years and older was carried out to assess self-reported influenza testing in the last 30 days for the 2021‐2022 (n=335,964), 2022‐2023 (n=334,584), and 2023‐2024 (n=269,624) seasons. This table included the numbers and proportions of those who reported testing for influenza in each season. Survey weights to reflect US census targets were applied to proportions only.

Demographics		Season											
		2021‐2022				2022‐2023				2023‐2024			
		Tested for influenza^a^		Only tested for influenza^b^		Tested for influenza^a^		Only tested for influenza^b^		Tested for influenza^a^		Only tested for influenza^b^	
		n	Weighted % (MOE^c^)	n	Weighted % (MOE)	n	Weighted % (MOE)	n	Weighted % (MOE)	n	Weighted % (MOE)	n	Weighted % (MOE)
Gender													
	Male	4463	4.4 (4.3‐4.6)	392	0.4 (0.4‐0.4)	8041	9.3 (9.1‐9.6)	1238	1.4 (1.3‐1.5)	5146	8.8 (8.5‐9.1)	1062	1.8 (1.7‐2.0)
	Female	6976	3.9 (3.7‐4.0)	544	0.3 (0.3‐0.4)	12,862	8.8 (8.6‐9.0)	2128	1.5 (1.4‐1.6)	8280	8.9 (8.7‐9.2)	1612	1.9 (1.7‐2.0)
	Not listed or non-conforming	294	5.0 (4.3‐5.8)	—^d^	—	591	11.4 (10.2‐12.6)	—	—	454	11.2 (9.7‐12.7)	—	—
Race													
	White	5639	3.1 (3.0‐3.2)	378	0.2 (0.2‐0.3)	9733	7.2 (7.0‐7.4)	1432	1.1 (1.1‐1.2)	6006	6.9 (6.6‐7.1)	1023	1.3 (1.2‐1.4)
	Black	2798	6.8 (6.5‐7.1)	265	0.7 (0.6‐0.8)	4982	13.9 (13.4‐14.4)	842	2.2 (2.0‐2.4)	3441	14.2 (13.6‐14.8)	719	3.1 (2.8‐3.4)
	Hispanic	2298	6.1 (5.8‐6.4)	221	0.6 (0.5‐0.7)	4176	12.5 (12.0‐13.0)	711	2.1 (1.9‐2.3)	2611	11.8 (11.2‐12.4)	578	2.5 (2.3‐2.8)
	Asian	462	3.4 (3.0‐3.8)	—	—	748	7.4 (6.8‐8.1)	181	1.7 (1.4‐2.0)	523	7.8 (6.9‐8.7)	182	2.5 (1.9‐3.0)
	No answer	536	4.7 (4.2‐5.2)	—	—	7236	10.1 (9.5‐10.7)	1216	1.6 (1.4‐1.9)	9228	10.7 (9.9‐11.5)	2077	2.5 (2.1‐2.9)
Education													
	Did not complete high school	684	5.7 (5.1‐6.2)	—	—	1359	12.4 (11.5‐13.3)	251	2.3 (1.9‐2.8)	1034	12.1 (11.0‐13.2)	252	3.3 (2.7‐3.9)
	High school or General Educational Development diploma	2971	5.2 (5.0‐5.5)	270	0.5 (0.4‐0.6)	5630	10.9 (10.5‐11.3)	696	1.9 (1.7‐2.1)	3917	10.6 (10.1‐11.1)	804	2.2 (2.0‐2.4)
	Associate’s degree	1335	4.2 (3.9‐4.5)	104	0.3 (0.3‐0.4)	2253	9.0 (8.5‐9.5)	330	1.3 (1.1‐1.5)	1409	8.5 (7.9‐9.1)	291	1.8 (1.5‐2.1)
	Some college	2569	4.0 (3.8‐4.2)	222	0.4 (0.3‐0.4)	4625	9.0 (8.6‐9.3)	727	1.3 (1.2‐1.5)	2939	9.0 (8.5‐9.4)	541	1.7 (1.5‐1.9)
	College graduate	2409	2.7 (2.6‐2.8)	175	0.2 (0.2‐0.2)	4464	6.5 (6.2‐6.7)	699	1.0 (0.9‐1.1)	2645	6.3 (5.9‐6.6)	523	1.2 (1.1‐1.4)
	Postgraduate degree	1765	2.4 (2.3‐2.6)	118	0.2 (0.1‐0.2)	3163	5.7 (5.4‐6.0)	475	0.9 (0.8‐1.0)	1936	5.7 (5.4‐6.1)	346	1.0 (0.9‐1.2)
Age (years)													
	13‐17	201	4.2 (3.4‐5.0)	—	—	396	10.1 (8.7‐11.5)	101	2.1 (1.4‐2.8)	352	10.8 (9.0‐12.6)	131	3.9 (2.7‐5.0)
	18‐24	1086	5.4 (5.1‐5.8)	110	0.5 (0.4‐0.7)	1735	10.8 (10.1‐11.5)	334	2.0 (1.7‐2.4)	1184	10.7 (9.8‐11.6)	271	2.4 (2.0‐2.8)
	25‐34	2319	6.2 (5.9‐6.5)	170	0.5 (0.4‐0.6)	3992	12.8 (12.3‐13.3)	571	1.8 (1.6‐2.0)	2297	11.1 (10.5‐11.7)	419	1.9 (1.6‐2.1)
	35‐44	2471	4.9 (4.7‐5.2)	164	0.4 (0.3‐0.4)	4533	10.8 (10.4‐11.2)	713	1.8 (1.6‐2.0)	2820	10.5 (10.0‐11.0)	518	1.9 (1.7‐2.1)
	45‐54	2392	3.9 (3.7‐4.1)	185	0.3 (0.3‐0.4)	4040	8.3 (8.0‐8.7)	588	1.3 (1.1‐1.4)	2689	8.7 (8.2‐9.1)	473	1.6 (1.4‐1.8)
	55‐64	1857	2.8 (2.7‐3.0)	142	0.2 (0.2‐0.3)	3724	6.9 (6.6‐7.2)	607	1.1 (1.0‐1.2)	2432	7.2 (6.8‐7.5)	505	1.5 (1.3‐1.7)
	65 and older	1407	2.3 (2.1‐2.4)	175	0.3 (0.3‐0.4)	3074	5.6 (5.4‐5.9)	537	1.0 (0.9‐1.1)	2106	5.7 (5.4‐6.1)	440	1.2 (1.1‐1.4)
Income (US $)													
	Under 15,000	2002	7.1 (6.7‐7.5)	217	0.8 (0.7‐0.9)	3640	15.0 (14.4‐15.6)	536	2.3 (2.0‐2.5)	2319	14.2 (13.5‐14.9)	426	2.7 (2.3‐3.0)
	Between 15,000 and 29,999	1782	5.4 (5.1‐5.8)	162	0.5 (0.4‐0.6)	2999	12.0 (11.5‐12.5)	527	2.2 (2.0‐2.5)	1863	11.7 (11.0‐12.4)	350	2.2 (1.9‐2.5)
	Between 30,000 and 49,999	1879	4.4 (4.2‐4.7)	158	0.4 (0.3‐0.5)	3247	9.5 (9.1‐9.9)	496	1.4 (1.2‐1.5)	1993	9.7 (9.2‐10.2)	411	2.1 (1.8‐2.3)
	Between 50,000 and 74,999	1687	3.7 (3.5‐3.9)	145	0.3 (0.2‐0.4)	2897	8.0 (7.6‐8.4)	471	1.3 (1.1‐1.4)	1706	7.8 (7.3‐8.3)	367	1.7 (1.5‐1.9)
	Between 75,000 and 99,999	1206	3.2 (3.0‐3.4)	—	—	2041	7.4 (7.0‐7.8)	325	1.3 (1.1‐1.4)	1160	7.1 (6.6‐7.6)	236	1.6 (1.4‐1.9)
	Between 100,000 and 150,000	1288	2.8 (2.6‐2.9)	—	—	2175	6.3 (6.0‐6.6)	344	1.1 (1.0‐1.2)	1209	6.1 (5.7‐6.6)	224	1.3 (1.1‐1.5)
	Over 150,000	1287	2.8 (2.6‐3.0)	—	—	2183	6.3 (5.9‐6.6)	295	0.9 (0.8‐1.0)	1211	6.0 (5.6‐6.4)	190	1.2 (1.0‐1.4)
	No answer	601	3.3 (3.0‐3.6)	—	—	7693	7.1 (6.6‐7.6)	1388	1.4 (1.2‐1.7)	10348	7.2 (6.6‐7.9)	2375	1.9 (1.9‐1.6)
Insurance													
	Plan through your spouse, parent, or employer	4180	3.2 (3.0‐3.3)	265	0.2 (0.2‐0.3)	6536	7.1 (6.9‐7.4)	947	1.1 (1.0‐1.2)	3604	7.0 (6.7‐7.3)	626	1.4 (1.3‐1.6)
	Other (please specify)	819	4.5 (4.2‐4.9)	—	—	789	9.2 (8.4‐10.0)	166	1.8 (1.5‐2.2)	—	—	—	—
	Plan you purchased yourself	1515	4.7 (4.4‐5.0)	141	0.4 (0.3‐0.5)	2446	9.1 (8.7‐9.6)	404	1.5 (1.4‐1.7)	1478	9.8 (9.2‐10.4)	298	1.9 (1.6‐2.2)
	Medicare	2263	4.2 (4.0‐4.4)	242	0.4 (0.4‐0.5)	4470	9.5 (9.2‐9.8)	706	1.6 (1.4‐1.7)	2843	9.4 (9.0‐9.8)	545	1.9 (1.7‐2.1)
	Medicaid or Medical	1659	6.7 (6.3‐7.1)	130	0.6 (0.4‐0.7)	3357	14.4 (13.8‐15.0)	487	2.1 (1.9‐2.3)	1985	13.2 (12.5‐13.9)	376	2.6 (2.3‐3.0)
	TRICARE	262	4.8 (4.1‐5.5)	—	—	501	11.6 (10.4‐12.8)	—	—	232	8.9 (7.5‐10.4)	—	—
	Not covered by health insurance	777	4.7 (4.3‐5.1)	—	—	1112	8.6 (8.0‐9.2)	181	1.3 (1.1‐1.6)	753	7.9 (7.2‐8.7)	162	1.6 (1.3‐2.0)
	No answer	257	4.7 (4.0‐5.5)	—	—	7664	9.3 (8.6‐10.1)	1406	1.9 (1.6‐2.3)	10,914	9.3 (8.6‐9.9)	2517	2.3 (2.0‐2.7)
Industry													
	Health care	2166	4.3 (4.1‐4.5)	147	0.3 (0.3‐0.4)	3851	9.7 (9.4‐10.1)	555	1.4 (1.3‐1.6)	2145	9.1 (8.6‐9.6)	424	1.9 (1.6‐2.0)
	Non–health care	6269	4.2 (4.1‐4.3)	478	0.3 (0.3‐0.4)	10,772	9.0 (8.8‐9.3)	1745	1.5 (1.4‐1.6)	6192	8.9 (8.6‐9.2)	1203	1.7 (1.6‐1.9)

a "Tested for influenza” includes all individuals who answered “Yes” to “In the past 30 days, have you been tested for influenza (flu)?”

b “Only Tested for influenza” includes only individuals who answered “Yes” to “In the past 30 days have you been tested for influenza (flu)?” and excluded an answer of “Yes” to “In the past 30 days, have you been tested for COVID-19?”

c MOE: Margin of Error.

d Suppressed values are indicated by “- -“ for groups with an N lower than 100.

Overall, positive results to influenza tests were low, ranging from 8.8% (1019/11,733) in 2021‐2022 to 12.0% (3228/26,875) in 2022‐2023 and 12.8% (2811/21,892) in 2023‐2024. Across seasons, the 25‐ to 34-year age group was the largest proportion of influenza positive results (27.0%, 241/1019; 28.6%, 631/2811; and 22.0%, 334/2811; respectively), followed by the subsequent 35‐ to 44-year age group (17.9%, 220/1019; 18.9%, 639/2811; and 19.0%, 407/3228; respectively). A full output of weighted proportions of influenza positive results among influenza testers by age is located in Table S4 in Multimedia Appendix 1.

### Multivariate Logistic Regressions

**Figure 1.**
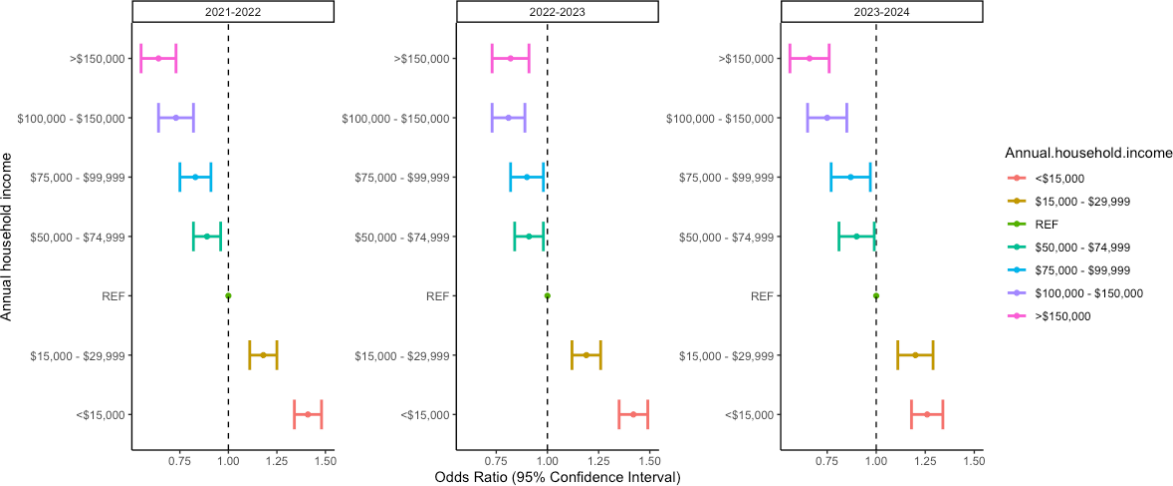
AOR for likelihood to have been tested for influenza by income, controlling for demographics and likelihood to test for COVID-19. An online cross-sectional study of US residents aged 13 years and older was carried out to assess self-reported influenza testing in the last 30 days for the 2021‐2022 (n=335,964), 2022‐2023 (n=209,646), and 2023‐2024 (n=269,624) influenza seasons. A weighted multivariate logistic regression was used to assess likelihood of influenza testing by income when controlling for demographics and COVID-19 testing. In all three seasons, those with lower incomes were observed to have a higher likelihood of testing for influenza, and those with higher incomes were less likely. AOR: adjusted odds ratio.

Likelihood to have been tested for influenza in the past 30 days decreased consistently as income increased, when adjusting for other variables. Those below the reference annual household income (US $30,000 and US $49,999) had higher odds of having been tested for influenza than those with higher incomes (Figure 1). As household income increased, the odds of having been tested for influenza decreased. This trend was observed consistently across the 2021‐2022, 2022‐2023, and 2023‐2024 influenza seasons, with slightly variant magnitudes (Figure 1). Those with an income under US $15,000 were most associated (adjusted odds ratio [AOR] 1.41, 95% CI 1.34‐1.48; AOR 1.42, 95% CI 1.35‐1.49; and AOR 1.25, 95% CI 1.18‐1.34; respectively) with being tested for influenza, and those with an income over US $150,000 were least associated (AOR 0.64, 95% CI 0.55‐0.86; AOR 0.82, 95% CI 0.73‐0.91; and AOR 0.66, 95% CI 0.56‐0.76; respectively) with being tested for influenza in the influenza seasons of 2021‐2022, 2022‐2023, and 2023‐2024. Complete results of the regressions can be found in Table S5 in Multimedia Appendix 1.

## Discussion

### Principal Findings

Our objective for this study was to assess who among an online, cross-sectional survey was tested for influenza between 2021‐2024. In each of the included influenza seasons, only a small amount of the population reported testing for influenza. We found higher proportions of lower income, lower educational attainment, and Black and Hispanic individuals among those who self-reported being tested for influenza in the past 30 days. Furthermore, those who made under US $15,000 a year were the most likely to report being tested for influenza, and those who made over US $150,000 were the least likely, across all 3 seasons. The observation of lower income and higher likelihood of influenza testing differs from other studies, which have found higher income individuals generally have perceived better access and uptake of respiratory disease testing, particularly for COVID-19 [10,21].

Increases in influenza testing over the study period were observed, though levels remained small compared to the overall sample. Advances in diagnostics, such as at-home and multiplex testing, may contribute to increases in testing for influenza in the 2023‐2024 season [7,8]. Here we found individuals who did not complete high school, made under US $15,000, and had income-assisted insurance had higher proportions of influenza testing. One potential explanation is that the population testing for influenza may need evidence of a health care visit, positive test result, or diagnosis to receive sick time or paid time off benefits. It is documented that people without paid sick leave benefits are less likely to receive influenza vaccination, use emergency care, or visit a health care professional when sick with respiratory illness [22,23]. This population is also likelier to include younger individuals, females, Hispanic individuals, those with a lower educational level, and those in certain occupations like blue-collar or farm jobs [23]. Our finding that lower income individuals are testing more may also be indicating that higher income counterparts have flexibility with their sick leave, such as remote work options [24-27]. Another contributing factor could be that populations known to experience severe influenza may be tested more due to higher hospitalization rates, existing comorbidities, or other factors leading to more interaction with health care during an influenza infection than other population groups [4].

Adjusting our regression model to account for health care worker status and COVID-19 testing further supports these findings as it accounts for those who may also require influenza testing for work. Similarly, adjusting for COVID-19 testing in the past 30 days indicates these trends were not driven by COVID-19 testing during the pandemic. Overall levels of influenza testing were consistently lower than those of COVID-19 testing throughout the study. Furthermore, the population reporting testing for influenza varied from the observed population of COVID testers during a similar time, which tended to be higher income among other demographics [5,6,10,11]. This difference further highlights that there are potential economic differences and impacts in testing for either influenza or COVID-19 during 2021‐2024. The overlap of our study and the pandemic does introduce nonnormal behaviors, interest, and access to testing. Further study of these trends would be insightful to see if they persist.

This study encompasses strengths in its methodology that lend to the validity of the observed results. Data used for this study was collected from a previously validated, nationally representative survey, allowing us to generalize the observed results [10,17,18,20]. This validation and the weighting scheme that enables findings to encompass census targets minimizes bias about those who participated differing from the US population broadly. The inclusion of data spanning 3 influenza seasons further supports the empirically observed trends as they were consistent across time. Furthermore, the study period captures any potential differences season-to-season and any changes due to changes in the COVID-19 pandemic [28].

This study is also subject to limitations. First, being cross-sectional, the trends observed are insights into different individuals at certain points in time, rather than longitudinally assessing a consistent cohort each season. This means that our insights are broader and not assessing specific individuals’ influenza testing and income over time. In addition, access to reliable internet is required for conducting the survey so populations that do not have reliable access are not captured. Similar to many health surveys, older women with higher income and higher educational attainment were more likely to complete the survey. Furthermore, health surveys generally capture an audience who may be more health-minded than the general population. The survey may also be subject to misclassification as participants are asked not only to self-report but to recall testing that occurred in the last 30 days and not an immediate time frame. Moreover, participants may not know what illnesses they were tested for, particularly in a clinical setting.

### Conclusion

As public health continually seeks to improve the prevention and mitigation of respiratory diseases, it is vital that we understand trends in who is testing for disease, and the implications for surveillance and response. Different access and uptake of testing by disease may be influenced by economic factors like employment, income, and benefits. These differences are important to understand for future public health efforts, such as modifying messaging around influenza testing, increasing affordable access to influenza testing, and creating policies to ease the burden of testing across illnesses when it is required to receive sick leave. Further studies are needed to understand the full scope of social and economic factors influencing testing behavior for respiratory diseases in the United States. In addition, studying differences across respiratory, rather than pan-respiratory diseases, may provide insights that support targeted interventions. As public health continues to strive for more robust disease mitigation and response, it becomes increasingly important not only to understand differences in access to testing, but disparities in who has the need for testing and official diagnoses and the implications on the health care system and disease surveillance overall.

## Supplementary material

10.2196/76459 Multimedia Appendix 1 Additional tables.

## Abbreviations

AOR: adjusted odds ratio
ILI: influenza-like illness
MMWR: Morbidity and Mortality Weekly Report
ONM: Outbreaks Near Me
