# Supplementary material for: Assessment of Influenza Testing Distribution in the United States for the 2021-2022, 2022-2023, and 2023-2024 Influenza Seasons: Online Cross-Sectional Study
Source: JMIR Public Health Surveill. 2025 Aug 26;11:e76459. doi: 10.2196/76459 (PMC12379747; doi:10.2196/76459)
Supplement: Multimedia Appendix 1 [file publichealth-v11-e76459-s001.docx]

**Appendix**

*Supplemental Table 1: Start and End Dates of Each Influenza Season*

| *Season* | *Start Date* | *End Date* |
| --- | --- | --- |
| 2021-2022 | 10-04-2021 | 03-31-2022 |
| 2022-2023 | 10-03-2022 | 03-31-2023 |
| 2023-2024 | 10-02-2023 | 03-31-2024 |

*An online cross-sectional study of U.S. residents thirteen and older was done to assess self-reported influenza testing in the last thirty days for the 2021-2022 (N=335,964), 2022-2023 (N=334,584), and 2023-2024 (N=269,624 ) seasons. Supplemental Table 1 details the exact date ranges included for each influenza season. Start dates were determined by MMWR week 40 and end date was truncated to March 31^st^ as that was the last date of data collection in 2024.*

*Supplemental Table 2: Weighted Industry Percentages for All Industry Options Present in the Survey*

|  | | Season | | | | | |
| --- | --- | --- | --- | --- | --- | --- | --- |
| Industry | | **2021-2022** | | **2022-2023** | | **2023-2024** | |
|  | | N | Weighted% | N | Weighted % | N | Weighted % |
| Advertising & Marketing  Agriculture  Airlines & Aerospace  Automotive  Business Support & Logistics  Construction  Consulting & Research  Education  Entertainment & Leisure  Finance & Financial Services  Food & Beverages  Government  Hospitality  Pharmaceuticals  Insurance  Legal  Manufacturing  Nonprofit  Personal service  Retail & Consumer Goods  Real Estate  Technology  Transportation & Delivery  Utilities & Energy  No Answer | 3562  2558  1896  4020  3897  9720  3951  24221  3488  9601  10522  13380  3497  2879  3971  4068  10779  5717  12368  4206  10280  6313  3830  11582  11582 | | 1.64  1.44  0.82  2.41  1.91  5.87  1.25  7.36  1.55  3.52  6.88  5.07  1.67  1.05  1.51  1.30  5.62  2.42  2.81  6.66  1.65  4.00  3.67  1.94  5.93 | 1869  1388  980  2003  2204  5198  2002  12451  1922  4627  6276  6900  2151  1444  1823  2041  5502  4137  3226  5945  2092  4559  3444  1863  7000 | 1.62  1.44  0.86  2.30  1.98  5.94  1.25  7.48  1.62  3.25  7.66  4.97  1.91  1.00  1.34  1.23  5.52  2.62  2.84  5.89  1.54  3.59  3.58  1.77  5.64 | 1493  1305  846  1821  1822  4975  1586  10615  1547  3226  5310  5642  1900  1001  1258  1631  4584  3531  2748  2644  1581  3261  2944  1424  5891 | 1.65  1.53  0.86  2.53  1.92  6.71  1.24  8.07  1.66  2.78  7.50  5.20  1.95  0.90  1.22  1.30  5.80  2.91  2.90  5.72  1.45  3.28  3.73  1.64  4.24 |

*An online cross-sectional study of U.S. residents thirteen and older was done to assess self-reported influenza testing in the last thirty days for the 2021-2022 (N=335,964), 2022-2023 (N=334,584), and 2023-2024 (N=269,624 ) seasons. Supplemental Table 2 displays the full output of industry that was groups as “non-healthcare” in the study. Survey weights to reflects U.S. census targets were applied to proportions only.*

*Supplemental Table 3: Weighted Industry Flu Testing Percentages for All Industry Options Present in the Survey*

| Season | *2021-2022* | | *2022-2023* | | *2023-2024* | |
| --- | --- | --- | --- | --- | --- | --- |
|  |  | *Tested for influenza^a^* |  | *Tested for influenza^a^* |  | *Tested for influenza^a^* |
| Industry | N | Weighted % | N | Weighted % | N | Weighted % |
|  |  |  |  |  |  |  |
| Advertising & Marketing | 179 | 6.7 | 262 | 12.5 | 144 | 11.7 |
| Agriculture | -- | -- | 170 | 9.2 | -- | -- |
| Airlines and Aerospace | -- | -- | 103 | 8.0 | -- | 9.5 |
| Business Support & Logistics | 180 | 5.6 | 265 | 10.2 | 167 | 9.6 |
| Construction | 198 | 5.6 | 337 | 11.5 | 174 | 10.7 |
| Consulting and Research | 363 | 4.4 | 701 | 9.9 | 410 | 8.8 |
| Education | -- | -- | 158 | 8.6 | 102 | 8.3 |
| Entertainment and Leisure | 821 | 4.0 | 1438 | 9.5 | 898 | 9.5 |
| Finance & Financial Services | 119 | 4.3 | 248 | 10.9 | 132 | 8.6 |
| Food & Beverages | 290 | 3.3 | 457 | 8.1 | 227 | 8.0 |
| Government | 561 | 5.8 | 952 | 10.9 | 568 | 10.7 |
| Hospitality | 426 | 3.8 | 671 | 6.9 | 387 | 7.4 |
| Healthcare | 161 | 5.0 | 271 | 9.9 | 180 | 12.1 |
| Pharmaceuticals | -- | -- | 132 | 7.1 | -- | -- |
| Insurance | 105 | 2.9 | 168 | 6.0 | -- | -- |
| Legal | -- | -- | 184 | 7.9 | 109 | 7.8 |
| Manufacturing | 327 | 3.7 | 532 | 7.2 | 349 | 8.4 |
| Nonprofit | 220 | 3.3 | 431 | 8.5 | 258 | 7.8 |
| Personal Service | 238 | 4.7 | 404 | 9.7 | 224 | 8.8 |
| Retail & Consumer Goods | 472 | 4.3 | 687 | 9.1 | 339 | 7.0 |
| Real Estate | 105 | 2.6 | 201 | 7.6 | 108 | 7.4 |
| Technology | 249 | 2.8 | 396 | 7.5 | 256 | 9.3 |
| Transportation & Delivery | 238 | 4.0 | 419 | 8.3 | 226 | 7.4 |
| Utilities & Energy | 107 | 3.1 | 146 | 6.1 | 109 | 7.8 |
| No answer | 471 | 5.1 | 1039 | 10.1 | 517 | 9.9 |

*An online cross-sectional study of U.S. residents thirteen and older was done to assess self-reported influenza testing in the last thirty days for the 2021-2022 (N=335,964), 2022-2023 (N=334,584), and 2023-2024 (N=269,624 ) seasons. Supplemental Table 3 displays the full output of industry that was groups as “non-healthcare” among those who reported testing for influenza in each season. Survey weights to reflects U.S. census targets were applied to proportions only.*

*^a “^Tested for influenza” includes all individuals who answered “Yes” to “In the past 30 days, have you been tested for influenza(flu)?”*

*Suppressed values are replaced with “--“ due to low N’s (less than 100).*

*“Only tested for influenza” which excludes those who reported testing for COVID-19 in the “Tested for influenza” group is excluded from this table as the values were suppressed.*

*Supplemental Table 4: Influenza positive results, by age, among those who reported testing for influenza*

|  | Season | | | | | | |
| --- | --- | --- | --- | --- | --- | --- | --- |
| Age | **2021-2022** | | | **2022-2023** | | **2023-2024** | |
|  | N | | Weighted% | N | Weighted % | N | Weighted % |
| 13-17  18-24  25-34  35-44  45-54  55-64  65 and over | | --  116  241  220  191  121  103 | --  17.6  27.0  17.9  11.6  8.4  8.8 | --  246  631  639  431  411  308 | --  14.2  28.6  18.9  10.7  9.8  9.8 | --  177  334  407  346  134  362 | --  14.7  22.0  19.0  13.5  4.7  15.3 |

*An online cross-sectional study of U.S. residents thirteen and older was done to assess self-reported influenza testing in the last thirty days for the 2021-2022 (N=335,964), 2022-2023 (N=334,584), and 2023-2024 (N=269,624 ) seasons. Supplemental Table 3 displays the number and weighted proportion of a self-reported positive results to an influenza test, by age group, among those who tested. Survey weights to reflects U.S. census targets were applied to proportions only.*

*^a^ Values for 13-17 are suppressed due to low N’s (less than 100).*

*Supplement 5: Multivariate Regression Output of Assessment of Likelihood to Influenza Test by Income*

| Demographics |  |  | | Season | | | | | | | | |
| --- | --- | --- | --- | --- | --- | --- | --- | --- | --- | --- | --- | --- |
|  |  | 2021-2022 | | | | 2022-2023 | | | 2023-2024 | | | |
| Gender |  | AOR | 95% CI | | p-value | N | 95% CI | p-value | | N | 95%CI | p-value |
|  | Male  Female | REF  0.82 | --  0.78-0.86 | | --  <0.001 | REF  0.80 | --  0.76-0.84 | --  <0.001 | | REF  0.84 | --  0.79-0.89 | --  <0.001 |
| Race |  |  |  | |  |  |  |  | |  |  |  |
|  | White  Black  Hispanic  Asian  No Answer | REF  1.67  1.43  1.01  1.34 | --  1.62-1.72  1.37-1.49  0.91-1.11  1.21-1.47 | | --  <0.001  <0.001  <1.0  <0.001 | REF  1.58  1.46  1.08  1.13 | --  1.53-1.63  1.40-1.52  0.98-1.18  1.01-1.25 | --  <0.001  <0.001  <1.0  <0.05 | | REF  1.92  1.63  1.61  1.34 | --  1.86-1.98  1.56-1.70  1.49-1.73  1.26-1.42 | --  <0.001  <0.001  <0.001  <0.001 |
| Education |  |  |  | |  |  |  |  | |  |  |  |
|  | Did not complete high school  High school or G.E.D  Associate’s degree  Some college  College graduate  Post graduate degree | 1.32  REF  0.89  0.79  0.59  0.54 | 1.23-1.41  --  0.81-0.97  0.73-0.85  0.52-0.66  0.45-0.63 | | <0.001  --  <0.01  <0.001  <0.001  <0.001 | 1.04  REF  0.88  0.82  0.64  0.53 | 0.95-1.13  --  0.81-0.95  0.77-0.87  0.58-0.70  0.52-0.46 | <1.0  --  <0.01  <0.001  <0.001  <0.001 | | 1.04  REF  0.87  0.86  0.59  0.53 | 0.94-1.14  --  0.78-0.96  0.79-0.93  0.51-0.67  0.43-0.63 | <1.0  --  <0.01  <0.001  <0.001  <0.001 |
| Age |  |  |  | |  |  |  |  | |  |  |  |
|  | 13-17  18-14  25-34  35-44  45-54  55-64  >65 | 1.06  1.24  1.09  REF  0.83  0.73  3.51 | 0.98-1.14  1.17-1.31  1.02-1.16  --  0.75-0.91  0.64-0.82  2.51-4.51 | | <1.0  <0.001  <0.05  --  <0.001  <0.001  <0.05 | 0.99  1.03  1.09  REF  0.83  0.69  0.49 | 0.88-1.10  0.95-1.11  1.03-1.15  --  0.67-0.90  0.61-0.77  0.41-0.57 | <1.0  <1.0  <0.01  --  <0.001  <0.001  <0.001 | | 1.38  1.10  1.0  REF  0.88  0.74  0.60 | 1.25-1.51  1.01-1.19  0.92-1.08  --  0.80-0.96  0.64-0.84  0.52-0.68 | <0.001  <0.05  <1.0  --  <0.01  <0.001  <0.001 |
| Insurance |  |  |  | |  |  |  |  | |  |  |  |
|  | Plan through your spouse, parent, employer  Other (please specify)  Plan you purchased yourself  Medicare  Medicaid or Medical  TRICARE  Not covered by health insurance | REF  1.10  1.22  1.43  1.10  2.10  0.91 | --  1.00-1.20  1.15-1.29  1.37-1.49  1.13-1.27  1.96-2.24  0.82-1.00 | | --  <0.001  <0.001  <0.001  <0.001  <0.001  <0.001 | REF  1.10  1.22 1.43  1.20  2.10  0.91 | --  1.00-1.20  1.15-1.29  1.37-1.49  1.13-1.27  1.96-2.24  0.82-1.00 | --  <1.0  <0.001  <0.001  <0.001  <0.001  <1.0 | | REF  --  1.34  1.32  1.27  1.83  0.82 | --  --  1.26-1.42  1.25-1.39  1.19-1.35  1.65-2.01  0.72-0.92 | <0.001  <0.001  <0.001  <0.001  <0.001 |
| Industry |  |  |  | |  |  |  |  | |  |  |  |
|  | Healthcare  Non-healthcare | 1.07  REF | 1.02-1.12  -- | | <0.05  -- | 1.07  REF | 1.02-1.12  -- | <0.05  -- | | 1.09  REF | 1.01-1.17  -- | <0.05  -- |
| COVID-19 Testing |  |  |  | |  |  |  |  | |  |  |  |
|  | COVID-19 Tested  Not COVID-19 Tested | REF  0.03 | --  0.00-0.08 | | --  <0.001 | REF  0.03 | --  0.00-0.08 | <0.001 | | REF  0.02 | REF  0.00-0.07 | --  <0.001 |

*An online cross-sectional study of U.S. residents thirteen and older was done to assess self-reported influenza testing in the last thirty days for the 2021-2022 (N=335,964), 2022-2023 (N=334,584), and 2023-2024 (N=269,624 ) seasons. A weighted multivariate logistic regression was used to assess likelihood of influenza testing by income when controlling for demographics and COVID-19 testing. Supplement 4 displays the full output of the covariates in the regression model.*
